# Relevance acquisition through motivational incentives: Modeling the time-course of associative learning and the role of visual features

**DOI:** 10.1162/imag_a_00162

**Published:** 2024-05-08

**Authors:** Francesco Grassi, Louisa Kulke, Alex Lepauvre, Anne Schacht

**Affiliations:** Department for Cognition, Emotion and Behavior, Georg-August University Göttingen, Göttingen, Germany; Developmental Psychology with Educational Psychology, University of Bremen, Bremen, Germany; Neural Circuits, Consciousness, and Cognition Research Group, Max Planck Institute for Empirical Aesthetics, Frankfurt am Main, Germany; Donders Institute for Brain, Cognition and Behaviour, Radboud University Nijmegen, Nijmegen, the Netherlands

**Keywords:** associative learning, motivational relevance, EEG, pupil dilation, modeling neural changes across time, pseudowords

## Abstract

Motivational relevance associated with symbolic stimuli impacts both neural and behavioral responses, similar to visual stimuli with inherent emotional valence. However, the specific effects of associated relevance on early sensory stages and lexico-semantic processing of these stimuli remain unclear, particularly considering the role of low-level visual features in relevance acquisition. To address these issues, we employed an associative learning paradigm in which we manipulated visual features, but not the stimuli themselves. The study (N = 48) included a learning phase, where pseudowords were associated with either gain, loss, or neutral outcomes. This was followed by a test phase the next day, involving an old/new decision task, in which stimuli were presented in either the same or a different font. During both phases, pupil responses and event-related brain potentials (P1, Early Posterior Negativity (EPN), Late Positive Complex (LPC), P3) were measured. Stronger pupil responses and increased neural activation in early visual encoding (P1) and lexico-semantic processing (EPN) were observed during relevance acquisition, particularly for loss associations. After relevance acquisition, the most substantial effect on modulating lexico-semantic processing was observed for gain associations, as evidenced by both behavioral responses and neural activity. During the test phase, exposure to incongruent visual features of the stimuli influenced the same processes that were observed during relevance acquisition. Notably, these effects of visual feature congruence were independent of those of associated motivational relevance. These results highlight the dynamic nature of motivational relevance effects, revealing differential effects observed during acquisition and the test phase, as well as between earlier perceptual processing and later neural and behavioral responses.

## 1 Introduction

Visual stimuli play a crucial role in our perception and cognition, and their processing is influenced by various factors, including emotional and motivational relevance. Certain visual stimuli, such as facial expressions of emotion, receive prioritized processing and rapid allocation of attention due to their inherent emotional and motivational relevance (Anderson, 2013). This prioritization allows for elaborate appraisal and appropriate responses to potentially rewarding or dangerous events. Even neutral objects can acquire motivational relevance through associative learning processes, leading to modulations of behavioral and neural responses similar to those observed for stimuli of inherent relevance (Hammerschmidt, Kulke, et al., 2018;Hammerschmidt et al., 2017;Rossi et al., 2017;Schacht et al., 2012;Ziereis & Schacht, 2023a). Such associations can endow these objects with relevance, making them capable of capturing attention and affecting our cognitive and emotional responses.

Associations of neutral stimuli with motivationally relevant outcomes, such as monetary gain, have been consistently learned faster than associations with neutral outcomes (Bayer et al., 2019;Grassi et al., 2023;Hammerschmidt, Kulke, et al., 2018;Kulke et al., 2019;Rossi et al., 2017). Furthermore, some of these studies report that the advantages persisted even when the association was no longer relevant, resulting in faster reaction times (Grassi et al., 2023;Hammerschmidt, Kulke, et al., 2018), and better recognition (Rossi et al., 2017) compared to neutral stimuli. Overall, the behavioral effects of associated motivational relevance appear to indicate processing advantages in terms of sensory encoding, memory, and response production. At the neural level, the impact of associated motivational relevance has been investigated using event-related potentials (ERPs). Several studies have shown that the association of motivational relevance can modulate ERP responses to a variety of visual stimuli, including faces (Hammerschmidt, Kagan, et al., 2018;Hammerschmidt, Kulke, et al., 2018;Hammerschmidt et al., 2017) and symbols (Bayer et al., 2017;Rossi et al., 2017). Interestingly, similar neural modulations have also been recently reported when the acquired relevance came not from motivational incentives but from association with inherently emotional stimuli, as, for example, vocal affect bursts (Ziereis & Schacht, 2023b).

Notably, research has suggested that neural responses to visual stimuli, such as faces (Hammerschmidt et al., 2017) or Chinese characters (Schacht et al., 2012), can be influenced as early as 100 ms (P1) by the learned associations of these stimuli with motivational incentives. Similarly, even earlier ERP components, such as the C1, have been found to be enhanced in response to line gratings associated with threat-related pictures (Stolarova et al., 2006) or to meaningless symbols associated with monetary loss (Rossi et al., 2017). These early effects have been functionally linked to modulations of attentional processes associated with low-level visual features (Grassi et al., 2023;Rossi et al., 2017).

Written words represent a unique category of visual objects since the association of their visual form with their semantic and emotional content is arbitrary and must be acquired through learning (Bayer et al., 2019;Schacht & Sommer, 2009b). The time-course of word processing has been a topic of debate. Previous evidence suggests that the first 200 ms primarily reflect orthographic processing, with lexico-semantic features being accessed at later stages (for a review, seeBarber & Kutas, 2007). Conversely, other studies indicate that lexico-semantic features can be accessed already within the initial 200 ms after stimulus onset (Davis et al., 2019;Feldman et al., 2015;Hauk et al., 2012;Shtyrov & Macgregor, 2016). Using existing words as stimulus material, however, makes it difficult to distinguish the specific contributions of perceptual and semantic features due to their inherent and predetermined association.

To disentangle the role of visual and semantic features in the processing of symbolic stimuli, researchers often employ pseudowords. Pseudowords are visually similar to words but lack semantic content. They have been used to examine the acquisition of motivational relevance through associative learning (Bayer et al., 2019;Kulke et al., 2019), as well as classical (Montoya et al., 1996) or evaluative conditioning (Fritsch & Kuchinke, 2013;Kuchinke et al., 2015). The association of pseudowords with negative motivational relevance can influence early ERP components, such as the P1 (Bayer et al., 2019;Grassi et al., 2023;Kulke et al., 2019), which is assumed to reflect the perceptual encoding of visual stimuli (Di Russo et al., 2003;Hillyard & Anllo-Vento, 1998). These findings suggest that low-level visual features may convey the motivational relevance of symbolic stimuli, irrespective of their semantic content. Associations of relevance to low-level visual features have also been proposed as the mechanism underlying early effects observed in the processing of visual stimuli with inherent emotional valence (for review:Pourtois & Vuilleumier, 2006;Vuilleumier, 2005). Although progress has been made in understanding early effects of acquired motivational relevance on the processing of symbolic stimuli, evidence regarding the magnitude and direction of these effects remains inconsistent. For example, studies have reported differential effects of associated positive and negative motivational relevance on the P1 component depending on whether real words with semantic meaning or relevance-associated pseudowords were used (Kulke et al., 2019). In addition, later ERP components, such as the Early Posterior Negativity (EPN) and the Late Positive Complex (LPC), have been reliably shown to be modulated by motivationally relevant symbolic stimuli (e.g.,Bayer et al., 2012;Citron, 2012;Hinojosa et al., 2010;Schacht & Sommer, 2009a). The EPN component is thought to reflect the automatic capture of attention by relevant stimuli, and its modulation has been predominantly observed in response to meaningful words of emotional content (Bayer et al., 2012;Palazova et al., 2011;Schacht & Sommer, 2009b), but not in response to stimuli lacking semantics (Rellecke et al., 2011;Schacht et al., 2012). However, recent evidence has challenged this notion:Grassi et al. (2023)found EPN modulations in response to meaningless symbolic stimuli that had acquired motivational relevance through associative learning, suggesting that the “meaning” of symbolic stimuli may extend beyond their semantic content and depend on the demands of a particular task.

Additionally, the P3 component, considered to reflect the activity of a fronto-temporal-parietal network involved in top-down attentional focus and memory processes (Polich, 2007), has also been a subject of interest in previous studies examining symbolic stimuli. In this context, increased P3 amplitudes have been observed in response to familiar stimuli compared to novel stimuli (Friedman, 1990;Johnson et al., 1985;Karis et al., 1984). Modulations of the P3 component have been reported in response to emotional words (Bernat et al., 2001), as well as positively and negatively associated words and symbols (Fritsch & Kuchinke, 2013;Rossi et al., 2017). However, it is worth noting that the evidence regarding modulations of the P3 component by associated relevance is mixed, as some studies reported no effects (Montoya et al., 1996), or effects limited to positively associated stimuli (Schacht et al., 2012).

In addition to ERPs, pupillary responses have been extensively used as a valuable measure of cognitive and emotional processing (Beatty, 1982). Several studies have observed increased pupil responses to emotional pictures (Bradley et al., 2008;Hess, 1965) and auditory stimuli (Jürgens et al., 2018;Partala & Surakka, 2003;Riese et al., 2014). This effect, found for both positive and negative stimuli, is thought to be driven by the arousing nature of the stimuli rather than their specific emotional valence (Bradley et al., 2008;Janisse, 1974). Only a few studies have investigated pupil responses in the context of symbolic stimuli of emotional content. The findings from these studies are somewhat inconclusive. In response to real words of emotional content, some studies reported no significant effects on pupil responses (Kuchinke et al., 2007), while others demonstrated enhanced pupil responses to positive words (Võ et al., 2008) or to low-arousing words (Bayer et al., 2011). This apparently different pattern of effects during the processing of written words compared to pictorial or auditory stimuli with emotional content has been interpreted as automatic facilitation, with less cognitive efforts required for the processing of highly arousing words (Bayer et al., 2011;Võ et al., 2008). However, it is essential to note that at least one study using abstract symbolic stimuli that had acquired relevance through reinforcement learning showed increased pupil responses to symbols associated with loss, but not to symbols associated with gain (Pulcu & Browning, 2017).

In summary, the precise mechanisms and temporal dynamics underlying the processing of symbolic stimuli are still not fully characterized. In particular, it remains unclear what specific contribution low-level visual features play in acquiring emotional and motivational relevance, and to what extent acquired relevance impacts behavioral, physiological, and neural responses. The primary objective of this study was to investigate the impact of visual features on the associative learning of symbolic stimuli. To examine the role of visual features in associative learning of motivational relevance, rather than semantic context, the study employed a design in which only specific visual features, namely the font type of pseudowords, were altered between learning and recognition. The two font types used differed maximally in their visual shape. We hypothesized that if the effects of associated relevance are related to visual features, they would disappear when the previously learned pseudowords are presented in a different font type, that is, with different low-level visual features.

Previous studies have consistently shown that positive associations are learned faster than negative or neutral associations (e.g.,Bayer et al., 2019;Hammerschmidt et al., 2017;Rossi et al., 2017). However, there is a gap in the literature regarding the investigation of how motivational relevance affects behavioral and neural responses during the acquisition of these associations (cf.Grassi et al., 2023). To fill this gap, the present study aimed to model associative learning mechanisms at the behavioral, pupillary, and neural levels. It also aimed to investigate the effects of visual features by analyzing responses to stimuli presented in a font type identical to or different from the one associated with them. For this purpose, we conducted a within-subject study, directly following the procedure ofKulke et al. (2019), with a learning phase on one day, in which pseudowords were associated with gain, loss, or neutral outcome, and a test phase on the following day.

For the learning phase, we hypothesized that gain and loss would exhibit faster associative learning, as well as heightened pupil response and enhanced amplitudes of the P1 and LPC components compared to the zero-outcome association. Since the pseudowords were initially neutral and novel, we also hypothesized that the enhanced P1 and LPC amplitudes in response to the relevance-associated stimuli would result from a relative increase in these component amplitudes across the learning phase trials compared to the zero-outcome stimuli. For the test phase, we expected that the enhanced pupil size, P1 and LPC responses observed during the learning phase would persist, even though the associated monetary outcome was no longer relevant to the task. Based on our hypothesis of enhanced learning of gain- and loss-associated stimuli, we hypothesized that recognition of these stimuli during the test phase would be facilitated, resulting in faster reaction times and higher accuracy compared to stimuli previously associated with a neutral outcome.

Given the functional significance of the P3 component in attentional focus and memory processes, we hypothesized that stimuli presented in the same font as in the learning phase would elicit increased P3 amplitudes during the test phase compared to stimuli presented in a novel font. Exploratorily, we also examined the potential effects of monetary outcome association on EPN amplitudes during both the learning and test phases. To investigate the role of low-level visual features in associative learning, we further explored the potential interaction effect between previously associated monetary outcomes and font congruence on all ERP components during the test phase.

The study involved an initial group of 24 participants. After a preliminary analysis, a replication study was preregistered, which included 24 additional participants as well as the combined dataset of 48 participants (preregistration:https://osf.io/xuntq/). However, the present study partially deviated from the preregistered hypotheses and analysis plan. A detailed rationale for these deviations is provided in the Supplementary Materials (Sections S1 and S2).

## 2 Methods

### 2.1 Participants

Data were collected from 61 native German, right-handed, female participants with normal vision (maximum +/-1 dioptric) and no history of neuropsychological disorders. The sample size was determined to be at least 10 times the number of predictors used in the analysis. Thirteen participants were excluded due to poor data quality (i.e., more than 50 percent of the trials rejected during pre-processing,*n*= 6), to insufficient learning success (*n*= 6), or misunderstood instructions (*n*= 1). This resulted in a final sample of 48 participants between 18 and 27 years of age (*M*= 22.40,*SD*= 2.58). Participants were reimbursed with €8.50 per hour and an additional performance-based bonus, as described below.

### 2.2 Stimuli

The stimuli consisted of 408 disyllabic pseudowords that were created according to the phonological rules of the German language in the form of consonant-vowel-consonant-vowel, as used byBayer et al. (2019)andKulke et al. (2019). For each participant, 24 of the 408 stimuli were randomly selected as targets and the rest as distractors. Target stimuli were presented in two different fonts, “Brush Script Std” and “Courier new” font, and each distractor was presented in one of the two fonts. These two specific fonts were chosen based on a pilot study, in which they were rated as the most dissimilar of the 20 font types presented. The stimuli were created in Adobe Illustrator CS6 (v. CC 2017.1), and luminance was adjusted using the software Adobe Photoshop CS6 (v. 13.0.4). All stimuli were presented in black color, with a size of 45 pt on a light gray background. Assignment of the target pseudowords to each monetary category and to each font type was counterbalanced between participants.

### 2.3 Procedure

The study was approved by the local Ethics Committee of the Institute of Psychology at the University of Göttingen, and all participants gave informed consent prior to participation. The experimental phases took place in an EEG cabin, where participants sat in a height-adjustable chair with their chin resting in a height-adjustable chin rest. The experiment consisted of two phases: a learning phase and a subsequent test phase, delayed by one day. The experiment was built in Python (version 3.6.8) using the package PsychoPy (version 3.1.2;Peirce et al., 2019) and run using PyCharm (version 2018.3.5). All stimuli were presented centrally on a BenQ XL2411Z monitor at a viewing distance of 60 cm in front of the participants.

#### 2.3.1 Learning phase

**Fig. 1.**
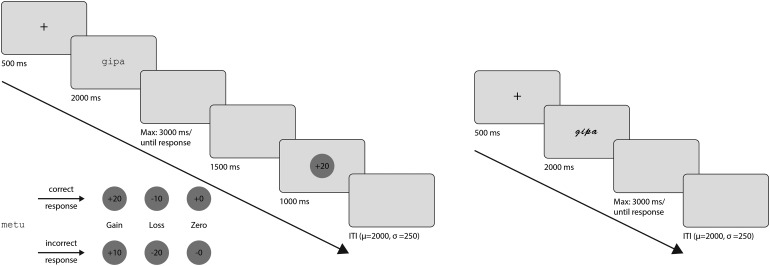
(Left) Trial scheme of the learning phase with feedback stimuli shown in accordance with the different outcome categories and participant response. (Right) Trial scheme of the test phase.

Upon arrival, participants completed a consent and demographic data questionnaire. In the learning phase, participants were presented with 24 target stimuli (each presented in one of the two fonts), one-third of which was associated with a reward, with a loss, or with a neutral outcome each. The assignment of individual target stimuli to the outcome condition was counterbalanced between participants, to ensure that outcome-related effects could not originate from potential differences in the visual form of psycholinguistic properties. Participants had to learn the association between the stimuli and the corresponding outcome category by pressing one of three keys on a standard German keyboard: left and right arrow for loss- and gain-associated pseudowords, down for neutral pseudowords (Fig. 1). The keys associated with gain and loss were counterbalanced across individuals. Participants started with a balance of 3 Euros. At the beginning of each trial, a fixation cross was presented at the center of the screen for 500 ms, followed by a stimulus presented for 2,000 ms. If no response was made during stimulus presentation, a blank screen was visible for a maximum of 3,000 ms or until the response. In each case, another blank screen followed for 1,500 ms. After participants responded, a feedback stimulus was displayed for 1,000 ms, informing participants to which outcome category the previously displayed stimulus belonged and whether their decision was correct: +20 or +10 was displayed for gain-associated stimuli if they were correctly or incorrectly categorized, respectively; -10 or -20 was displayed for loss-associated pseudowords if they were correctly or incorrectly categorized, respectively; and +0 or -0 was displayed following a zero-outcome stimulus if it was correctly or incorrectly categorized, respectively. The value displayed reflected the impact on the participant’s monetary balance in cents. If participants did not respond during the allotted time of 5,000 ms, -50 was displayed on the screen and 50 cents were subtracted from their account. After an inter-trial interval of 2,000 ms +/- 250 ms, the next trial began. The outcome categories of the stimuli were initially unknown to the participants; they had to learn the associations through a trial-and-error process. Learning was considered complete when participants correctly classified 48 pseudowords into their respective categories in a series of 50 pseudoword presentations. Once this criterion was met, the learning phase was terminated. The phase was divided into sub-blocks, with all 24 stimuli being presented once within each sub-block, in a randomized order. Participants could take a break between each sub-block.

#### 2.3.2 Test phase

On the following day, participants returned for the test phase. The 24 previously associated stimuli were presented and intermixed with 384 distractors. Participants had to determine whether the pseudoword was known or unknown from the previous phase, by pressing the left or right arrow, counterbalanced as known/unknown stimuli. Each of the 24 stimuli was presented in two font types: in the same font type as in the learning phase (referred to as the congruent font condition) and in the alternative font type (referred to as the incongruent font condition). The test phase consisted of 8 blocks. Within each block, the 48 target stimuli and the 48 distractors (randomly selected from the set of 386 distractor pseudowords without replacement, half in one font and the other half in the other font) were presented. Each distractor was presented only once during the entire phase. Participants could take a break between blocks. No feedback was given during the test phase.

### 2.4 EEG recording

During both phases, EEG was recorded using active Ag-Cl electrodes mounted on an Easy Cap EEG-cap fitted to the participant’s head according to the extended international 10–20 system (Pivik et al., 1993). External electrodes were applied bilaterally on the mastoids, outer canthi (HEOG), and below the eyes (VEOG). Common mode sense (CMS) was used as reference; driven right leg (DRL) as ground. The recorded signal was amplified using a Biosemi ActiveTwo AD-Box (24 bits; band pass filter 0,16 Hz to 100 Hz) and digitized at a sampling rate of 512 Hz. The electrodes offsets were kept between +/-25 mV.

### 2.5 Pupil size recording

The pupil size was continuously monitored throughout the phases using a desk-mounted Eye-tracker (EyeLink 1000, SR Research) recording at a sampling rate of 500 Hz. The device was calibrated for each phase by attaching artificial pupils with a set diameter of 5 mm to the participant’s closed eyelids to provide a reference for pupil size, and by having participants complete a 9-point calibration and validation routine to calibrate fixation position.

### 2.6 Data pre-processing

Preprocessing of the EEG data from the*test phase*was performed in Brain Vision Analyzer (V 2.1), following the procedures of (Kulke et al., 2019). Signals from external electrodes were removed from the dataset, the signal from the 64 scalp electrodes was re-referenced to the average reference, and the data were downsampled to 500 Hz based on spline interpolation. Zero phase shift Butterworth filters were applied: A low-pass filter with a high cutoff frequency of 40 Hz, a high-pass filter with a low cutoff frequency of 0.01591549 Hz, and a notch filter of 50 Hz were applied. All trials with correct responses were then epoched 200 ms prior to and 1,000 ms after stimulus onset. A 200-ms baseline was subtracted. Artifacts were identified as any time points exceeding +/-200 µV and rejected. The final sample of the test phase included an average of 56 trials for the gain–congruent condition (range: 36–64), 54 trials for the gain–incongruent condition (range: 29–64), 55.73 trials for the loss–congruent condition (range: 36–64), 55 trials for the loss–incongruent condition (range: 37–64), 55 trials for the zero-outcome–congruent condition (range: 36–64), 54 trials for the zero-outcome–incongruent condition (range: 37–64), and 338 trials for the distractors (range: 193–380). The data were finally averaged across trials for each outcome condition and each electrode independently.

The same pre-processing steps were performed in MATLAB (R2018a) for the data from*learning phase*, using the EEGLAB (version 2021.1;Delorme & Makeig, 2004), ERPLAB (version 8.10;Lopez-Calderon & Luck, 2014), and BioSig (version 3.7.9;Schölgl et al., 2011) toolboxes. After filtering, Independent Component Analysis was used to remove ocular artifacts, blinks, and channel noise from the data of the*learning phase*. Only epochs with correct responses were included in the analyses. Additionally, the mean amplitude of each ERP component was computed on a single trial basis and was then stored and used in later processing. After rejection, the dataset included an average of 81 trials for the gain condition (range: 28–257), 72 trials for the loss condition (range: 29–183), and 69 trials for the zero-outcome condition (range: 23–182). The pupil data were preprocessed in MATLAB. Artifacts were identified for each participant, separately for the learning and the test phases, as samples in which the difference in pupil size from the subsequent sample was greater than 0.1 mm, or in which the difference from the sample median exceeded 1 mm. Artifacts were removed, and missing data were interpolated using a linear interpolation based on the clean data samples. The data were epoched from 200 ms before to 3,000 ms after stimulus onset, with a 200-ms baseline subtraction. The data were then averaged for each participant and experimental condition.

### 2.7 Data analysis

#### 2.7.1 Behavioral and pupil data analysis

The behavioral data collected during the learning phase were analyzed using a Bayesian additive multilevel regression model for binomial responses (Fahrmeir et al., 2009) to determine the posterior distribution for the probability of correctly assigning the outcome category. The model was run using BayesX (version 3.0.2,Belitz et al., 2015), applying a Markov Chain Monte Carlo algorithm with 52,000 iterations, a burn-in period of 2,000, and a thinning parameter of 50. The input data consisted of correct trials per block and outcome category. To account for performance dependence, varying intercepts and slopes for participants across trials were included. Weakly informative priors were set to 𝛼 = 0.001 and*β*= 0.001, respectively. Time points at which the outcome conditions differed significantly were identified from the model estimates based on a criterion of non-overlapping 99% concurrent credible bands.

Analyses of the behavioral data from the test phase were performed in R (version 4.1.0;R Core Team, 2020). The effects of outcome condition, font congruence, as well as their interaction on reaction times and accuracy rates were estimated using Generalized Linear Mixed Models (GLMM;Baayen et al., 2008), fitted with the*lme4*package (version 1.1.-27;Bates et al., 2015). For reaction times, a GLMM with normal error structure was fitted with the function*lmer*. For accuracy rates, a GLMM with binomial error structure and logit link function was fitted via the function*glmer*. For both models, outcome condition, with the levels zero outcome, loss and gain, font congruence, with the levels congruent and incongruent, and their interaction were included as fixed effects. To keep the type I error rate at the 5% level, all identifiable random slopes were included (Barr et al., 2013;Schielzeth & Forstmeier, 2009), that is, outcome condition and font congruence within participant ID. Reaction times were log-transformed, and outlier greater than 2*SD*from the means were removed (Baayen & Milin, 2010). The overall effect of outcome condition, font congruence, and their interaction was tested via a full-null model comparison (Forstmeier & Schielzeth, 2011), where the null model lacked the main effects as well as their interaction but was otherwise identical to the full model. For reaction times, the significance of each level of the two factors and their interaction was tested by means of the Satterthwaite approximation (Luke, 2017), via the function*lmer*from the*lmerTest*package (version 3.1-3;Kuznetsova et al., 2017). These models were fitted once with zero outcome as the reference level of factor outcome condition, and again with gain as reference level, to test for a significant difference between gain and loss condition. Assumptions of normally distributed and homogenous residuals were assessed by visual inspection of the QQ plot of residuals (Field, 2005) and residuals plotted against fitted values (Quinn & Keough, 2002). The absence of collinearity issues was confirmed by refitting each model without the interaction term and calculating the Variance Inflation Factors, using the function*vif*from the*car*package (version 3.0-11;Fox & Weisberg, 2018). Model stability at the level of estimated coefficients and standard deviations was assessed by excluding one level of the random effects (i.e., subject) at a time and refitting the models (Nieuwenhuis et al., 2012), using a function kindly provided by Roger Mundry (University of Goettingen). Confidence intervals of the model estimates were calculated by parametric bootstrapping, using the function*bootMer*of the package*lme4*(N = 1,000 bootstraps).^1^

Pupil responses were quantified between 750 ms and 1,750 ms after stimulus onset. A GLMM with a normal error structure was fitted to test the effect of the outcome condition on the pupil data of the learning phase, and the effects of outcome condition, font congruence, and their interaction on the pupil data of the test phase.

#### 2.7.2 ERP analysis

The time windows and electrode clusters of interest for the P1, EPN, LPC, and P3 components were defined according toKulke et al. (2019): The P1 mean and peak amplitude at an occipital cluster (O1, O2, Oz) between 80 and 120 ms, the EPN mean amplitude at an occipito-parietal cluster (O1, O2, P9, P10, PO7, PO8) between 250 and 300 ms, the LPC mean amplitude at an occipito-parietal cluster (Pz, POz, PO3, PO4) between 400 and 600 ms, and the P3 mean amplitude at a centroparietal cluster (CPz, P1, Pz, P2) between 400 and 600 ms.

The preregistered study also included the analysis of the EPN component in an additional time window between 280 and 450 ms. However, upon inspection of the grand-averaged waveform and the topographical distribution of the ERP responses, the time window was adjusted to an interval between 200 and 300 ms. Results of this analysis are reported inSection S3of the Supplementary Materials.

Analysis of the ERP data was performed in R. Effects of outcome condition on the amplitudes of the P1, EPN, and LPC components in the learning phase were tested using additional LMMs, including outcome condition as a fixed effect and participant ID as a random intercept. Weights were added to account for the different number of trials from which the average ERP amplitudes were calculated after trial rejection. Normality assumptions, model stability, and confidence intervals were assessed as described above.

To further investigate the evolution of the associated outcome effect on ERP amplitudes during the learning process, a moving average of the single trials’ amplitude of each component was computed as follows: for each participant, 20 successive trials of the same outcome condition were averaged together, progressing from the first trials to the trial in which the participant reached the learning criterion, in an overlapping fashion (trial 1 to 20, 2 to 21, 3 to 22, etc.). A timestamp was computed for each averaged unit relative to the end of the learning phase: for each outcome condition, each averaged unit was indexed using zero-based indexing. The index of each unit was then divided by the last index number, creating a relative time scale for each participant ranging from 0 to 1 (0 being the first averaged unit, 1 being the last). Additional LMMs were then fitted including the covariate trial (as a relative averaged unit) and its interaction with outcome condition as fixed effects, and participant ID as a random effect. All identifiable random slopes were included, that is, outcome condition (manually dummy-coded and centered), trial, and their interaction within participant ID. Before fitting the models, the covariate trial was z-transformed to obtain a valid reference level for this predictor. We started with a maximal model, which included parameters for correlations between random intercepts and slopes. However, these were removed when they were estimated to have absolute values of essentially one and were therefore being unidentifiable (Matuschek et al., 2017). The overall effect of the interaction between outcome condition and trial was tested via a full null model comparison, where the null model lacked the interaction effect but was otherwise identical to the full model. Normality assumptions, absence of collinearity issues, model stability, and confidence intervals were assessed as described above.

The effects of associated monetary outcome and font congruence on the amplitudes of P1, EPN, LPC, and P3 components in the test phase were tested using LMMs, including outcome condition, font congruence, and their interaction as fixed effects, and outcome condition and font congruence within participant ID as random effects. The overall effect of the two main effects, and their interaction was tested via a full-null model comparison, where the null model lacked the main effects and the interaction effect but was otherwise identical to the full model. In the case of a nonsignificant interaction effect, reduced models including only the main effects of outcome condition and font congruence were fitted to better estimate the effect of the two factors. Normality assumptions, absence of collinearity issues, model stability, and confidence intervals were assessed as described above. For all ERP component models, the significance of each level of the factor outcome condition was first tested with zero-outcome as the reference level, and then again with gain as the reference level, to test for a significant difference between gain and loss levels.

## 3 Results

None of the LMMs showed any violation of assumptions or collinearity problems. All models showed acceptable stability. Models testing the effects of monetary outcome and font congruence on pupil response, as well as EPN and P3 amplitudes in the test phase, were fitted without correlation of random intercepts and slope, due to highly correlated random effects (≥.9). This resulted in only a small decrease in the model fit.

### 3.1 Learning phase

#### 3.1.1 Effects of monetary outcome on behavior

The learning curves are displayed inFigure 2A. Gain-associated pseudowords were learned faster and showed a significantly higher probability of correct classification compared to zero-outcome pseudowords between 2% and 49% of the learning phase, and compared to loss-associated pseudowords between 2% and 46% of the learning phase. The probability of correct classification during the learning phase did not differ between zero-outcome and loss-associated stimuli.

**Fig. 2.**
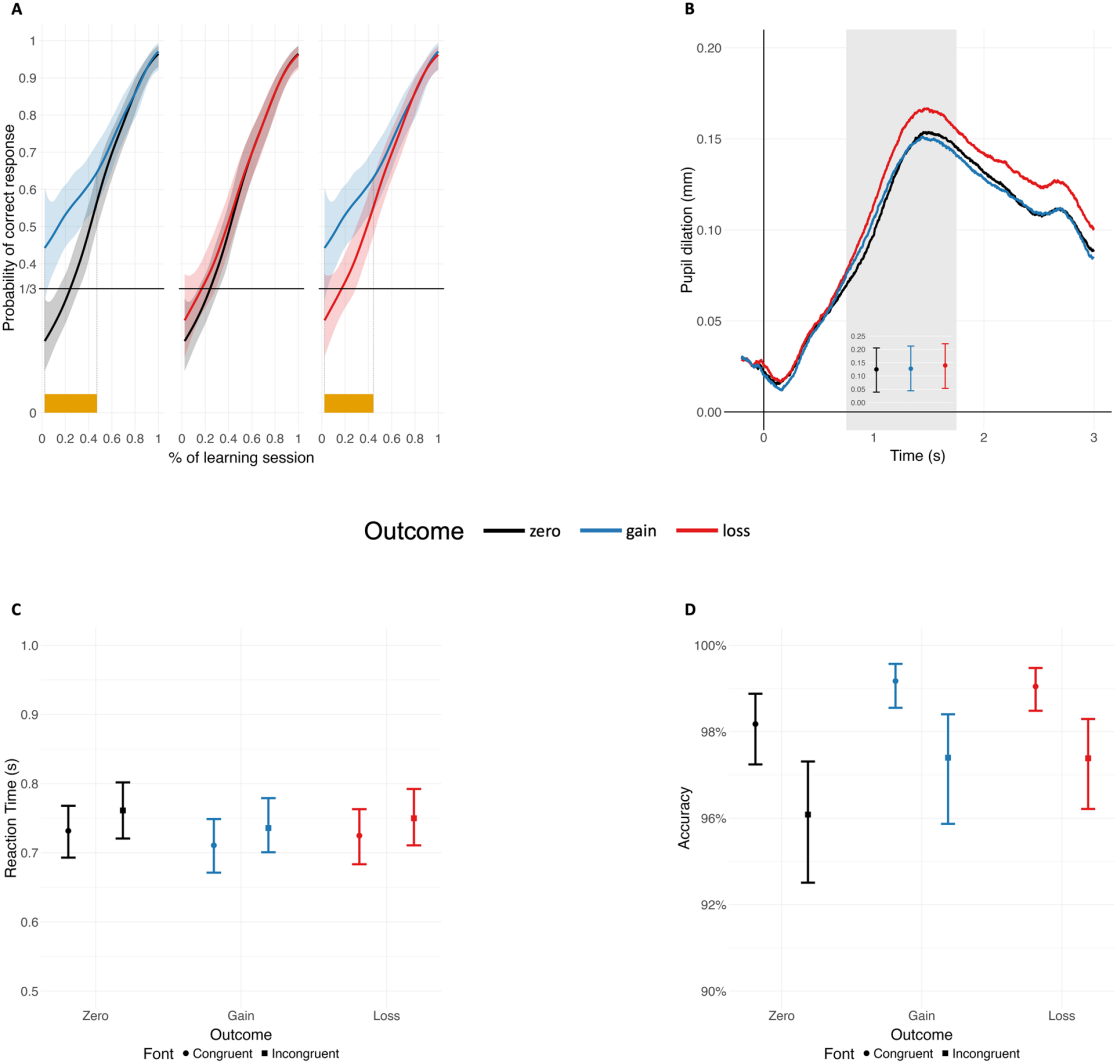
(A) Learning curves showing the mean probabilities of correctly assigning the outcome category correctly per outcome condition over the learning phase. Differences between outcome categories outside the 99% confidence bands are indicated by the orange segments along the x-axis. (B) Grand average pupil size time series during the learning phase. Inset shows estimated mean and 95% CI of pupil size for each outcome condition in the marked time window. (C) Estimated mean and 95 % CI of reaction time for each outcome condition and font congruence in the test phase. (D) Estimated mean and 95% CI of accuracy rates for each outcome condition and font congruence in the test phase.

#### 3.1.2 Effects of monetary outcome on pupil response

The model results are summarized inTable 1. Pseudowords associated with monetary loss elicited significantly larger pupil responses than both gain-associated and zero-outcome stimuli (*β*= 0.01,*SE*= 0.01,*p*= .009, and,*β*= 0.02,*SE*= 0.01,*p*= .002, respectively). There was no significant difference in pupil responses between gain-associated and zero-outcome stimuli (*p*> .05; seeFig. 2B).

**Table 1.** Summary of the LMMs regarding the effect of outcome association in the learning phase.

Predictors	R2	Estimate	Std. error	95% CI	t value	*p*
**Pupil Response**						
	.001					
intercept		0.13	0.02	0.04 0.21		^(1)^
outcome (gain) ^(2)^		0.00	0.01	-0.10 0.13	0.57	0.576
outcome (loss) ^(2)^		0.02	0.01	-0.10 0.13	3.27	0.002
**P1 (mean amplitudes)**						
	.001					
intercept		2.51	0.46	0.14 4.63		^(1)^
outcome (gain) ^(2)^		-0.11	0.15	-3.02 2.88	-0.76	0.456
outcome (loss) ^(2)^		-0.38	0.15	-3.28 2.35	-2.47	0.016
**P1 (peak amplitudes)**						
	.000					
intercept		4.13	0.46	1.83 6.46		^(1)^
outcome (gain) ^(2)^		-0.15	0.16	-3.22 2.92	-0.95	0.345
outcome (loss) ^(2)^		-0.42	0.16	-3.36 2.59	-2.58	0.012
**EPN**						
	.001					
intercept		-0.65	0.33	-2.83 1.50		^(1)^
outcome (gain) ^(2)^		-0.08	0.15	-2.95 2.84	-0.52	0.611
outcome (loss) ^(2)^		-0.41	0.16	-3.24 2.47	-2.63	0.011
**LPC**						
	.000					
intercept		3.55	0.37	0.87 6.27		^(1)^
outcome (gain) ^(2)^		0.01	0.21	-3.55 3.76	0.02	0.679
outcome (loss) ^(2)^		0.09	0.20	-3.93 3.88	0.42	0.983

(1) Not shown because of being of very limited interpretability.

(2) Comparison with the reference level (zero outcome).

#### 3.1.3 Effects of monetary outcome on mean ERP amplitudes

**Fig. 3.**
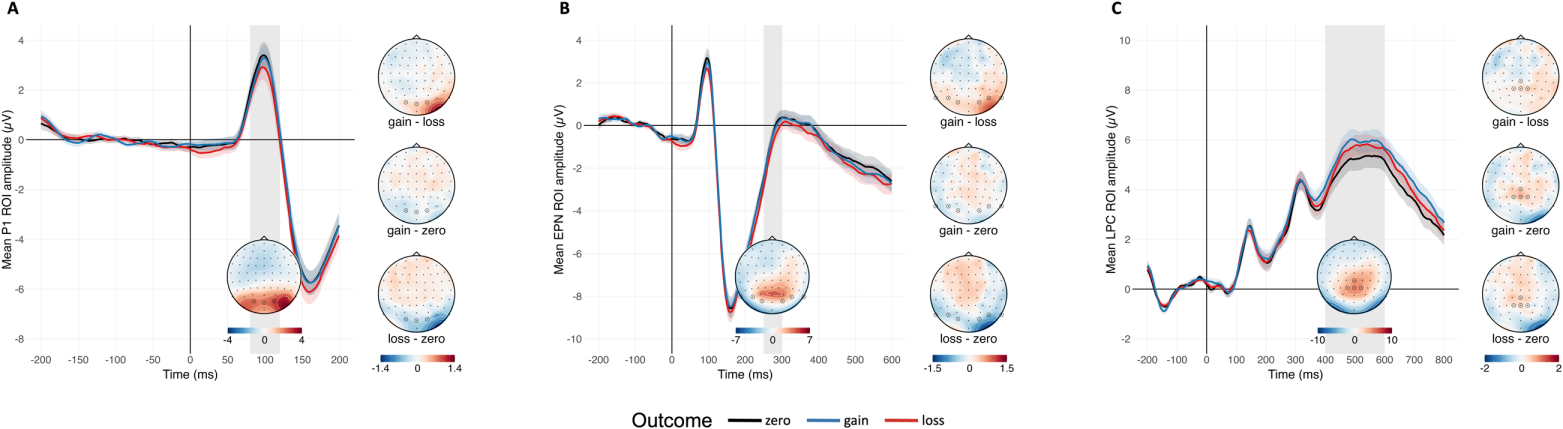
Grand-averaged ERPs during the learning phase, contrasted for zero, gain and loss outcomes, with Standard Mean Error (shaded areas), and corresponding scalp topographies of grand-averaged ERP across all conditions and of ERP differences between outcome conditions. (A) P1-ROI electrodes. (B) EPN-ROI electrodes. (C) LPC-ROI electrodes.

The model results are summarized inTable 1. Pseudowords associated with monetary loss elicited significantly lower**P1**amplitudes than zero-outcome stimuli (mean amplitudes:*β*= -0.38,*SE*= 0.15,*p*= .016; peak amplitudes:*β*= -0.42,*SE*= 0.16,*p*= .012). There was no significant difference between gain- and loss-associated or zero-outcome stimuli (Fig. 3A).

Loss-associated stimuli also resulted in more negative**EPN**amplitudes compared to both zero-outcome and gain-associated stimuli (*β*= -0.41,*SE*= 0.16,*p*= .011, and,*β*= -0.34,*SE*= 0.15,*p*= .030, respectively). Gain associations did not result in significant EPN modulations compared to zero-outcome stimuli (seeFig. 3B). No modulation of**LPC**amplitudes by monetary outcome associations was observed (all*p*s > .05; seeFig. 3C).

#### 3.1.4 Temporal development of relevance effects across learning

No significant interaction effect of associated monetary outcome and trial number was observed on the amplitudes of the P1, EPN, and LPC components (all*p*s > .05).

### 3.2 Test phase

The results of the test phase model are summarized inTables 2and3.

**Table 2.** Summary of the LMMs regarding the effect of outcome association and font congruence on behavioral and pupil responses in the test phase.

Predictors	R2	Estimate	Std. error	95% CI	t	*p*
**Reaction Times**						
	.014					
intercept		-0.31	0.01	-0.37 -0.26		^(1)^
outcome (gain) ^(2)^		-0.03	0.01	-0.09 0.04	-3.99	.000
outcome (loss) ^(2)^		-0.01	0.01	-0.07 0.05	-1.41	.167
congruence (incongr.)		0.04	0.00	-0.02 0.10	8.27	.000
outcome (gain) ^(2)^ * congruence (incongr.)		0.00	0.01	-0.10 0.09	-0.80	.431
outcome (loss) ^(2)^ * congruence (incongr.)		-0.01	0.01	-0.10 0.08	-0.89	.383
**Accuracy rates**						
	.059					
intercept		3.76	0.24	3.33 4.25		^(1)^
outcome (gain) ^(2)^		0.80	0.27	0.22 1.42	2.95	.003
outcome (loss) ^(2)^		0.66	0.25	0.14 1.17	2.62	.009
congruence (incongr.)		-0.79	0.15	-1.09 -0.52	-5.39	.000
outcome (gain) ^(2)^ * congruence (incongr.)		-0.38	0.19	-0.77 0.00	-1.98	.047
outcome (loss) ^(2)^ * congruence (incongr.)		-0.24	0.19	-0.65 0.14	-1.27	.206
**Pupil Response**						
	.000					
intercept		0.08	0.01	0.01 0.15		^(1)^
outcome (gain) ^(2)^		-0.01	0.01	-0.11 0.08	-1.55	.122
outcome (loss) ^(2)^		-0.01	0.01	-0.10 0.08	-1.00	.318
congruence (incongr.)		-0.01	0.01	-0.10 0.08	-1.11	.266
outcome (gain) ^(2)^ * congruence (incongr.)		0.00	0.01	-0.13 0.14	-0.04	.966
outcome (loss) ^(2)^ * congruence (incongr.)		0.00	0.01	-0.12 0.14	0.36	.719

(1) Not shown because of being of very limited interpretability.

(2) Comparison with the reference level (zero outcome).

**Table 3.** Summary of the LMMs regarding the effect of outcome association and font congruence on ERP amplitudes in the test phase.

Predictors	R2	Estimate	Std. error	95% CI	t	*p*
**P1 (mean amplitudes)**						
	.001					
intercept		2.68	0.40	1.85 3.43		^(1)^
outcome (gain) ^(2)^		-0.11	0.18	-0.45 0.22	-0.62	.539
outcome (loss) ^(2)^		-0.19	0.17	-0.49 0.15	-1.17	.250
congruence (incongr.)		-0.17	0.17	-0.49 0.17	-0.99	.327
outcome (gain) ^(2)^ * congruence (incongr.)		0.30	0.23	-0.13 0.76	1.31	.192
outcome (loss) ^(2)^ * congruence (incongr.)		0.31	0.23	-0.11 0.75	1.39	.172
**P1 (peak amplitudes)**						
	.001					
intercept		4.31	0.41	3.42 5.18		^(1)^
outcome (gain) ^(2)^		-0.07	0.20	-0.47 0.34	-0.36	.718
outcome (loss) ^(2)^		-0.21	0.19	-0.56 0.19	-1.07	.275
congruence (incongr.)		-0.23	0.19	-0.59 0.14	-1.26	.214
outcome (gain) ^(2)^ * congruence (incongr.)		0.25	0.26	-0.19 0.74	0.97	.331
outcome (loss) ^(2)^ * congruence (incongr.)		0.42	0.26	-0.10 0.94	1.61	.106
**EPN**						
	.010					
Intercept		-0.44	0.38	-1.20 0.33		^(1)^
outcome (gain) ^(2)^		-0.46	0.19	-0.83 -0.11	-2.40	.019
outcome (loss) ^(2)^		-0.66	0.19	-1.01 -0.29	-3.43	.001
congruence (incongruence)		-0.52	0.19	-0.90 -0.16	-2.81	.006
outcome (gain) ^(2)^ * congruence (incongr.)		0.29	0.26	-0.21 0.79	1.09	.282
outcome (loss) ^(2)^ * congruence (incongr.)		0.31	0.26	-0.18 0.84	1.20	.239
**LPC**						
	.005					
intercept		6.11	0.47	5.12 7.02		^(1)^
outcome (gain) ^(2)^		0.07	0.19	-0.29 0.45	0.37	.714
outcome (loss) ^(2)^		-0.05	0.20	-0.44 0.33	-0.28	.786
congruence (incongruence)		-0.47	0.20	-0.85 -0.08	-2.31	.023
outcome (gain) ^(2)^ * congruence (incongr.)		0.01	0.26	-0.46 0.50	0.03	.980
outcome (loss) ^(2)^ * congruence (incongr.)		0.10	0.26	-0.38 0.61	0.40	.693
**P3**						
	.006					
intercept		6.31	0.39	5.50 7.05		^(1)^
outcome (gain) ^(2)^		0.09	0.19	-0.26 0.46	0.46	.648
outcome (loss) ^(2)^		0.06	0.19	-0.30 0.48	0.33	.748
congruence (incongruence)		-0.26	0.19	-0.63 0.11	-1.39	.170
outcome (gain) ^(2)^ * congruence (incongr.)		-0.22	0.26	-0.75 0.26	-0.83	.412
outcome (loss) ^(2)^ * congruence (incongr.)		-0.18	0.26	-0.73 0.35	-0.68	.505

(1) Not shown because of being of very limited interpretability.

(2) Comparison with the reference level (zero outcome).

#### 3.2.1 Effects of associated monetary outcome and font congruence on behavior

Figure 2Cand2Ddisplay the estimated reaction times and accuracy rates in the test phase, for stimuli previously associated with monetary outcome, presented in either the same (congruent) or novel (incongruent) font, compared to the learning phase. The models showed acceptable stability, with no violations of assumptions, no collinearity issues, and no highly correlated random effects.

For reaction times, the full-null model comparison was statistically significant (likelihood ratio test:*χ2 = 70*.71,*df*= 5,*p*< .001). The interaction effect of monetary outcome and font congruence was not significant (*p*> .05). The reduced model fitted without the interaction effect showed significant main effects of both monetary outcome and font congruence. Reaction times to previously gain- and loss-associated pseudowords were faster compared to zero-outcome stimuli (*β*= -0.03,*SE*= 0.01,*p*< .001, and,*β*= -0.01,*SE*= 0.01,*p*= .051, respectively). Reaction times to stimuli previously associated with monetary gain were also faster than reaction times to loss-associated stimuli (*β*= -0.02,*SE*= 0.01,*p*= .016). Additionally, reaction times were faster when stimuli were presented in the congruent compared to the incongruent font (*β*= 0.04,*SE*= 0.00,*p*< .001).

The full-null model comparison of accuracy rates was also statistically significant (likelihood ratio test:*χ2 = 59*.62,*df*= 5,*p*< .001). The model revealed a significant interaction effect of monetary outcome and font congruence. Accuracy rates for stimuli previously associated with monetary gain were higher than for zero-outcome stimuli, but this difference was reduced when stimuli were presented in the incongruent compared to the congruent font (*β*= -0.38,*SE*= 0.19,*p*< .047).

#### 3.2.2 Effects of associated monetary outcome and font congruence on pupil response

The likelihood ratio test for the full-null model comparison did not show significant effects of the previously associated monetary outcome, font congruence, or their interaction on pupil responses (*χ2 = 7*.71,*df*= 5,*p*> .05).

#### 3.2.3 Effects of associated monetary outcome and font congruence on mean ERP amplitudes

For P1 amplitudes, the full-null model comparison did not reveal any significant effect of the previously associated monetary outcome, font congruence, or their interaction (likelihood ratio test for P1 mean amplitudes:*χ2 = 2*.78,*df*= 5,*p*> .05; likelihood ratio test for P1 peak amplitudes:*χ2 = 2*.74,*df*= 5,*p*> .05; seeFig. 4A).

**Fig. 4.**
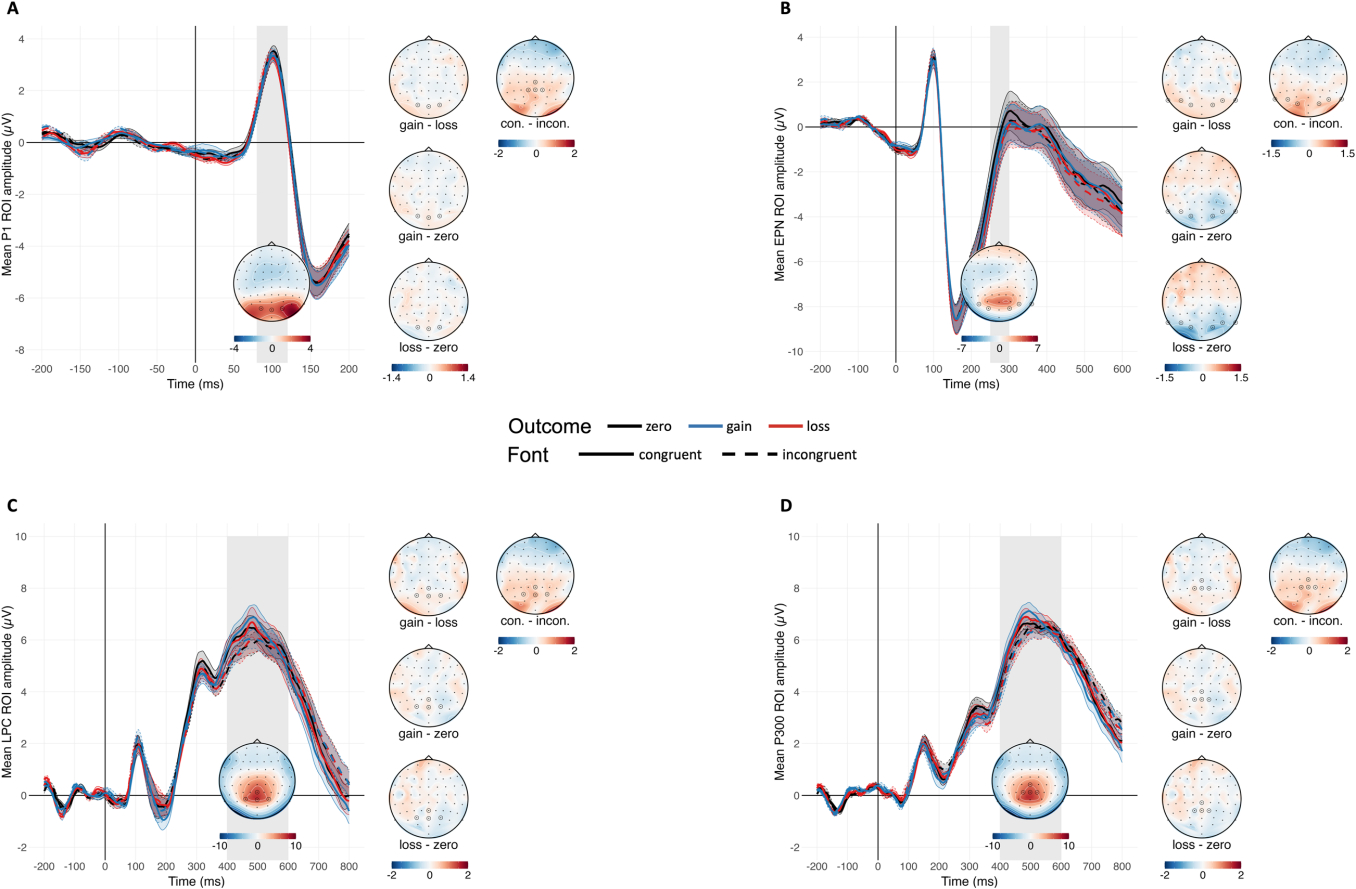
Grand-averaged ERPs during the test phase, contrasted for zero, gain and loss outcomes, and for congruent and incongruent font, with Standard Mean Error (shaded areas), and corresponding scalp topographies of grand-averaged ERP across all conditions, and ERP differences between conditions. (A) P1-ROI electrodes. (B) EPN-ROI electrodes. (C) LPC-ROI electrodes. (D) P3-ROI electrodes.

Likelihood ratio tests revealed significance for the full-null model comparisons in EPN, LPC, and P3 amplitudes (EPN:*χ2 = 22*.73,*df*= 5,*p*< .001; LPC:*χ2 = 11*.12,*df*= 5,*p*= .049; P3:*χ2 = 12*.07,*df*= 5,*p*= .034). However, none of the component amplitudes were significantly modulated by the interaction effect of outcome category and font congruence (all*p*s > .05).

For the EPN, the reduced model without the interaction term resulted in significantly more negative amplitudes for gain- and loss-associated pseudowords compared to zero-outcome stimuli (*β*= -0.32,*SE*= 0.14,*p*= .028, and*β*= -0.50,*SE*= 0.14,*p*= .001, respectively). In addition, EPN amplitudes were significantly more negative for pseudowords presented in the incongruent than in the congruent font (*β*= -0.32,*SE*= 0.11,*p*= .003;Fig. 4B).

The reduced models also showed a main effect of font congruence on LPC and P3 amplitudes (Fig. 4C, D), with lower amplitudes for incongruent compared to congruent fonts (*β*= -0.43,*SE*= 0.14,*p*= .003, and,*β*= -0.40,*SE*= 0.11,*p*= .001, respectively). All other effects did not reach significance (all*p*s > .05).

## 4 Discussion

### 4.1 The role of low-level visual features in associative learning of motivational relevance

The study aimed to investigate the role of low-level visual features of symbolic stimuli in the associative learning of motivational relevance. This was achieved by manipulating the congruence of the visual features (i.e., the font) between the learning and the test phases. If low-level visual features contributed to the acquisition of motivational relevance, we hypothesized that any relevance effects in the test phase would be limited to the stimuli presented with the same visual features as in the learning phase. First, an interaction effect was observed for accuracy rates in the test phase, where the retrieval advantage for stimuli previously associated with monetary gain was further boosted when the stimuli retained the same low-level visual features as during memory encoding. This finding highlights the role of low-level visual features in influencing memory retrieval, in addition to the advantage conferred by the association of motivational relevance. Second, the absence of an interaction effect of associated outcome and font congruence on ERP amplitudes in the test phase possibly does not invalidate our initial hypothesis. We believe that such a conclusion would be overly simplistic, as the results from the test phase present a more nuanced picture than anticipated.

Most of the ERP components analyzed in the test phase, namely P1, LPC, P3, showed no modulation by the associated outcome, regardless of its interaction with font congruence. In the absence of a main effect, therefore, it is not possible to draw strong conclusions about the specific role of low-level visual features in the association of motivational relevance, at least with respect to the neural processes underlying such ERP components.

The EPN component was unique in being modulated by both associated outcome and font congruence, allowing for a discussion of the reasons for the lack of interaction between the two factors. Similar modulations have been observed in studies using emotional words (Hinojosa et al., 2010;Hofmann et al., 2009;Palazova et al., 2011;Schacht & Sommer, 2009a,2009b;Scott et al., 2009), and a similar paradigm using word-like stimuli (Grassi et al., 2023). In the context of symbolic stimuli, emotion effects on the EPN have been observed to occur immediately after lexicality effects (Palazova et al., 2011;Schacht & Sommer, 2009b), and are thought to reflect the automatic capture of attention by the emotional content of linguistic stimuli (Bayer et al., 2012;Herbert et al., 2006;Kissler et al., 2007;Palazova et al., 2011;Schacht & Sommer, 2009a,2009b). The source dipole of the EPN component has been localized in the fusiform gyrus (Schacht & Sommer, 2009b), an area receiving both bottom-up projections from earlier visual areas (Distler et al., 1993) and top-down projections from more rostral temporal structures involved in the processing of semantic and phonological processing (Catani et al., 2003;Distler et al., 1993). Therefore, it has been proposed that the fusiform area serves as an interface between the abstract, form-invariant, representation of a stimulus and its higher-order, non-visual properties (Devlin et al., 2006;Grassi et al., 2023).

Our results align with this conceptual framework, as modulations of the EPN in the test phase would reflect the integration of higher-order information of associated motivational relevance with the form-invariant, that is, regardless of the font, representation of the pseudowords. Even without a significant interaction with associated relevance, our test phase results indicate that the congruence of low-level visual features impacted recognition of the stimuli, as evidenced by both neural and behavioral responses. Of particular interest are the enhanced EPN amplitudes observed for stimuli presented in the incongruent font. To the best of our knowledge, this is the first study investigating modulations of this component in response to manipulations of the visual features of symbolic stimuli. We propose that the enhanced EPN amplitudes to pseudowords in the incongruent font reflect the additional processing required to extract the abstract, form-invariant representation of a stimulus with novel low-level visual features.

It is important to compare this result with the absence of similar modulations of the P1 by font congruence. During the test phase, participants were directed to distinguish between old and new stimuli without any instructions on which information to use for the judgment. It is unclear whether they deemed items “old” based on the combination of pseudoword and font from the learning phase, or solely on the pseudoword itself. Participants may have focused on the pseudoword rather than the font, given that characters hold more significance than font in everyday word recognition. Therefore, the lack of P1 modulations by font congruence may be due to the irrelevance of processing such information for the task at hand.

Stimuli presented in the congruent font resulted in increased amplitudes in the LPC and P3 time windows. It is worth noting that the topographical distribution of the observed responses and their grand-averaged waveform do not clearly differentiate between LPC and P3 components. Previous studies that have employed similar paradigms (cf., e.g.,Bayer et al., 2019;Kulke et al., 2019;Rossi et al., 2017) interpreted modulations of centro-parietal positivities within the context of either LPC or P3 literature, based on the nature of the observed effects.

Due to the difficulties in distinguishing between closely related ERP components, particularly when they overlap in time and space, we take a cautious approach in interpreting our findings regarding LPC and P3 responses in our results. While we consider previous evidence on the effects of both components, we refrain from making definitive conclusions about the specific type of modulations observed. The modulations in the LPC/P3 time window are similar to the typical old/new effect reported in the literature (Bayer et al., 2019;Kulke et al., 2019;Rossi et al., 2017), with familiar stimuli eliciting larger LPC/P3 amplitudes compared to completely novel stimuli. However, other studies found that modulations of this component were influenced by the recollection of specific details of the stimulus (e.g., by manipulating the plurality of a word between the study and the test phase;Curran, 2000). Our findings expand on previous evidence, suggesting that the recollection mechanisms that contribute to ERP modulations in the LPC/P3 time window are also sensitive to modifications of the low-level visual features of symbolic stimuli.

The facilitated recollection of stimuli in a font congruent with the learning phase enabled participants for easier discrimination of these stimuli from the new ones in the test phase. This was reflected in improved accuracy rates and reaction times, consistent with previous research indicating that familiar fonts enhance behavioral responses to words (Kuchinke et al., 2014). Behavioral responses further benefited from the association of monetary gain, and, to a lesser extent, monetary loss, regardless of font congruence. This confirms previous evidence indicating that arbitrary stimuli with acquired motivational relevance, particularly those associated with rewards, remain preferentially processed even when the association is no longer relevant to the task (Grassi et al., 2023;Kulke et al., 2019;Rossi et al., 2017;Schacht et al., 2012). This behavioral advantage for relevance-associated stimuli was not reflected in the modulations of the P1, or LPC/P3 components, nor in the pupil response.

The presence of P1 modulations when the acquired association is no longer relevant for the task is inconsistent across studies with similar paradigms (cf., e.g.,Bayer et al., 2019;Grassi et al., 2023;Kulke et al., 2019;Rossi et al., 2017;Schacht et al., 2012). The absence of such modulations in our test phase might be due to the rapid extinction of early sensory prioritization observed during the learning phase, as the previously associated monetary outcome was no longer relevant to maximize participants’ performance (Grassi et al., 2023). In addition, variations in stimulus material (e.g., words, pseudowords, symbols) and procedures could affect the strength of associations and ease of extinction in the test phase.

The lack of relevance effects in the LPC/P3 time window contributes to mixed evidence regarding later modulations in the context of associative learning. Our findings are consistent with some studies (Bayer et al., 2019;Hammerschmidt et al., 2017;Kulke et al., 2019), while others have reported modulations in this time window (Grassi et al., 2023;Hammerschmidt, Kagan, et al., 2018;Hammerschmidt, Kulke, et al., 2018;Rossi et al., 2017;Ziereis & Schacht, 2023b). This inconsistency emphasizes the potential importance of task specifications and stimulus material (cf.Ziereis & Schacht, 2023a), and further research is needed to clarify these factors.

Finally, the absence of effects of associated relevance on pupil dilation contrasts with previous literature on real words. For example,Võ et al. (2008)found enhanced pupil responses to previously encountered stimuli, and this effect was stronger for neutral than positive or negative words, suggesting that the emotional content of known words facilitated memory retrieval, reducing cognitive effort in response to emotionally salient stimuli. One possible explanation for this difference could be that our task was simpler, which may have reduced the impact of the additional processing benefit for stimuli associated with relevance. It is worth noting that in our study, participants had to learn 24 pseudowords, each presented multiple times, while Võ and colleagues used 180 words, each presented only once. The complexity of the task and repeated exposure to stimuli may have affected the processing advantage during memory retrieval. This requires further investigation in future studies.

### 4.2 Neural dynamics of associative learning

Prior to learning, the pseudowords were unfamiliar stimuli without any (emotional) meaning. Therefore, any observed effects of motivational relevance must have evolved during the association acquisition phase. The second objective of the present study was to examine the temporal development of the ERP modulations throughout the learning phase.

However, none of these components showed any interaction effects between trial and associated outcome. Only a few studies have explored the time-course of similar effects, withGrassi et al. (2023)being the only study, to our knowledge, that investigated these dynamics with symbolic stimuli. Similar to our findings, they also reported no interaction effects for P1 and EPN but observed an interaction for LPC, demonstrating a faster increase in component amplitudes for gain compared to zero outcomes throughout the learning phase. As suggested by the authors, one possible explanation for the lack of interaction effects in our study is that modulations of ERP component amplitudes occur very early in the learning process and remain stable across the learning phase. These nonlinear dynamics may be difficult to capture with the linear mixed models used in our analysis. This interpretation is further supported by the effect on learning speed, which was evident early in the learning phase. Another explanation could be that, unlikeGrassi et al. (2023), we only analyzed trials in the learning phase up to the learning criterion. It is possible that modulations of ERP amplitudes continued to evolve during the later part of the learning phase, even though learning was achieved based on task performance. Further research is needed to investigate the precise temporal dynamics of ERP modulations during associative learning.

Although we did not observe any dynamic changes in ERP amplitudes during the learning phase, our results clearly demonstrate that the associated motivational relevance had an impact on behavioral, pupillary, and neural responses. As previous literature suggests (Bayer et al., 2019;Grassi et al., 2023;Kulke et al., 2019;Rossi et al., 2017), we observed a dissociation between behavioral responses and early perceptual and attentional processing stages.

Our study’s behavioral findings demonstrate that participants learned gain-associated stimuli faster than loss-associated and zero-outcome stimuli, aligning with prior research using similar associative learning paradigms (Bayer et al., 2019;Grassi et al., 2023;Kulke et al., 2019;Rossi et al., 2017). This indicates that reward association enhances the processing and learning of symbolic stimuli. The absence of a similar advantage for loss-associated stimuli may stem from different neural processes involved in the encoding of reward and loss information (Kim et al., 2015;Wächter et al., 2009), with loss information being encoded up to 100 ms slower than reward information (Chapman et al., 2015). As observed in a previous study employing a similar learning paradigm (Grassi et al., 2023), participants in the current study might have been tempted to respond as quickly as possible due to the risk of significant monetary loss for delayed responses. This time constraint could have particularly affected the slower encoding of loss.

Contrary to the behavioral advantage for gain associations, loss-associated stimuli elicited heightened pupil dilations compared to both zero-outcome and gain-associated stimuli. This finding contributes to mixed evidence regarding the effects of emotional and motivational relevance on pupil responses to symbolic stimuli. For example,Võ et al. (2008)reported increased pupil responses for positive compared to negative words, although the latter received higher arousal ratings.Bayer et al. (2011)provided evidence that pupillary responses to emotional words are primarily modulated by arousal, with low-arousing words inducing larger pupil responses compared to high-arousing ones. The authors suggested that high-arousing words are preferentially processed, thus requiring less cognitive effort, and resulting in reduced pupil responses. However, the increased pupil size for loss-associated stimuli in our study contradicts this interpretation. This discrepancy may stem from differences in the neural mechanisms underlying the motivational relevance of symbolic stimuli in our study compared to the emotional relevance of real words. Previous studies have demonstrated increased pupil responses to both positive and negative pictures, emphasizing the role of arousal over valence in influencing pupillary responses (Bradley et al., 2008). This aligns with observations for neutral faces with acquired motivational relevance in terms of monetary gain or loss (Hammerschmidt, Kagan, et al., 2018) and for meaningless symbols associated with loss but not with gain in a reinforcement learning paradigm (Pulcu & Browning, 2017).

At the neural level, it is significant to note the differing impacts of gain and loss associations on EPN amplitudes. Loss association led to amplitude changes during the learning phase, while gain association affected EPN amplitudes only during the test phase. This suggests that stimuli with negative relevance can swiftly capture attention and alter processing due to their higher arousal, whereas positive associations might necessitate longer exposure or memory consolidation. Additionally, the disparity in effects between learning and test phases could stem from variations in action disposition. In the learning phase, where the assessment of monetary outcomes was crucial, loss-associated stimuli might have induced greater arousal, driven by both loss avoidance and appetitive motivations. However, in the test phase, where associated outcomes were no longer relevant, the consequences of correct or incorrect responses did not differ between previously gain- and loss-associated stimuli, aligning with previous arguments (Rossi et al., 2017).

Finally, loss-associated stimuli had a significant impact on sensory encoding, as reflected in modulations of P1 amplitudes. Similar early effects have been reported for negative words (Bayer et al., 2012;Hofmann et al., 2009;Keuper et al., 2013;Scott et al., 2009), and it has been proposed that they arise from the lifelong association of the visual word forms with their emotional content (Keuper et al., 2013;Kuchinke et al., 2014;Rellecke et al., 2011). The preattentive, unconscious evaluation of aversive stimuli is thought to rely on bidirectional communications between the visual cortex and the amygdala (Morris et al., 1999;Pourtois et al., 2010,^2013^).Grassi et al. (2023)proposed that these initial effects could be due to feedback projections from the amygdala that improve the encoding of form-invariant representations of symbolic stimuli in the extra-striate visual cortex (Dehaene et al., 2005). It is important to note that in the present study, modulations of early sensory encoding were reflected in reduced P1 amplitudes for loss-associated compared to zero-outcome stimuli. This result is consistent with some previous literature (Kuchinke et al., 2014;Scott et al., 2009), while others have reported the opposite effect, with increased amplitudes for stimuli associated with loss (Bayer et al., 2019;Grassi et al., 2023;Rossi et al., 2017). This inconsistency has been attributed, at least partially, to methodological differences, including the choice of reference (Kuchinke et al., 2014). Additionally,Scott et al. (2009)proposed that a combination of arousal and valence might modulate the early perceptual encoding of symbolic stimuli, which could explain the reduced P1 amplitudes for loss-associated stimuli. Negative stimuli can cause increased arousal, which may result in a negative occipital wave. This can lead to decreased P1 amplitudes and increased N1 negativities. However, we cannot draw strong conclusions about the relative contribution of valence and arousal to the early sensory encoding of these stimuli since we did not measure the perceived arousal of the pseudowords after acquiring the association.

## 5 Conclusion

This study aimed to investigate whether and how low-level visual features impact associative learning with symbolic stimuli. The results revealed that the association of motivational relevance with visual features significantly influenced the processing of these stimuli, affecting behavioral, pupillary, and neural responses.

During relevance acquisition, loss associations elicited stronger pupil responses, along with increased neural activation in early visual encoding (P1) and lexico-semantic processing (EPN), while gain associations primarily impacted behavioral responses. Interestingly, after relevance acquisition, gain association had the most significant effect on lexico-semantic processing, evident in both behavior and neural activity. Moreover, exposure to incongruent visual features of the stimuli during the test phase further influenced these processes, independent of the effects of associated motivational relevance.

These findings collectively emphasize the dynamic nature of motivational relevance effects, varying across acquisition and test phases and between early perceptual processing and later neural and behavioral responses. While our results contribute to understanding associative learning effects, further research is needed to determine the shared neural mechanisms between motivational relevance in symbolic stimuli and emotional relevance in real words.

## Supplementary Material

Supplementary Material

## Data Availability

Data and the code behind analysis are publicly available at the Open Science Framework and can be accessed athttps://osf.io/cauxf/?view_only=f9eacc6b492e452ab3841a410bdd72f3. Raw EEG data are available upon request to the authors.

## Footnotes

1 Due to the different computation methods, the interpretation of Satterthwaite approximated*p*-values and bootstrapped Cis might differ.
