# Supplementary material for: Relevance acquisition through motivational incentives: Modeling the time-course of associative learning and the role of visual features
Source: Imaging Neurosci (Camb). 2024 May 8;2:imag-2-00162. doi: 10.1162/imag_a_00162 (PMC12247624; doi:10.1162/imag_a_00162)
Supplement: Supplementary Material [file imag_a_00162-supp.pdf]

Supplementary Materials

S1. Deviation from registered hypotheses

The study presented in the main text was initially conducted on a sample of 24 participants. After a preliminary analysis of this sample, we preregistered a replication of the study, with additional 24 participants (https://osf.io/xuntq/). From the preliminary analysis of the initial sample, we derived the following hypotheses:

1. P1 amplitudes would be modulated by relevance association.
2. If visual features play a role in the acquisition of relevance association, P1 amplitudes would be impacted by the font congruence (i.e., manipulation of visual features) in the test phase.
3. Pupil size would be modulated by relevance association.
4. If motivational relevance is associated to the low-level visual features of the stimuli, no relevance effect would be observed on the EPN and LPC amplitudes in the test phase, regardless of font congruence.
5. Congruent (old) font would elicit enhanced P300 amplitudes than incongruent (novel) font in the test phase.
6. During the learning phase, gain associations would be learned faster than zero-outcome ones.
7. P1 amplitudes would change over time during the learning phase, depending on relevance condition.
8. Gain associations would produce enhanced P1 amplitudes during the learning phase, compared to zero-outcome condition.

However, we were compelled to reconsider hypothesis 4 based on emerging evidence. Preregistered hypothesis 4 assumed that relevance was not associated with the visual features of the stimuli and therefore should not modulate later

processing stages reflected in EPN and LPC amplitudes. However, a study later conducted by our group (Grassi et al., 2023), used a similar experimental paradigm to associate motivational relevance with low-level visual features of symbolic stimuli. In this study, we observed significant modulation of both EPN and LPC amplitudes during the learning phase. Furthermore, modulations of LPC amplitudes persisted into the test phase.

In Grassi et al., (2023) we interpret EPN modulations as reflecting the integration of the visual representation of the stimulus with its higher-level, task-relevant representation. The study argues that modulations in this time-window can be expected if the task requires assigning the outcome category, which is the motivationally relevant information, to the stimulus' visual features. Given these new findings, we decided to deviate slightly from our preregistered hypothesis and to investigate modulations of the EPN component by relevance association in an exploratory manner, as we could not rule out the possibility of similar effects as observed in Grassi et al., (2023).

The same reasoning applies to LPC modulations. Although motivational relevance is associated with the low-level visual features of the stimulus, higher-level stages of stimulus processing may also be modulated by this relevance. Indeed, in this case we felt more confident to replace the preregistered hypothesis with the expectation of modulations of this component in both the learning and the test phase, as the same effects were observed in Grassi et al., (2023), as well as in other similar studies (e.g., Hammerschmidt, Kagan, et al., 2018; Hammerschmidt, Kulke, et al., 2018; Schacht et al., 2012; Ziereis & Schacht, 2023a).

Finally, we made a slight modification to preregistered hypothesis 6, which posited that gain associations would be learned faster than zero-outcome stimuli in the

learning phase. This adjustment was based on evidence from Grassi et al., (2023), which showed that loss associations were also learned faster than zero-outcome stimuli. We adjusted hypothesis 6 accordingly, expecting faster learning for both gain and loss associations.

S2. Deviation from registered analyses

The data analysis presented in the main text deviated from the preregistered study in some aspects. As mentioned in the previous section, the preregistered hypotheses were based on the preliminary analysis of the first sample of 24 participants. We intended to confirm the preliminary findings by analyzing a second sample of 24 participants, as well as the combined dataset of 48 participants. We believed that one preliminary result in particular required further validation: the differential change in P1 amplitude over time during the learning phase, depending on the associated relevance condition (preregistered hypothesis 7). To our knowledge, in fact, our study was among the first attempts to analyze such dynamical changes in ERP amplitudes during the course of relevance acquisition. In the current study, we have maintained the hypothesis as it is supported by the fact that any relevance effect on P1 amplitudes is expected to arise throughout the learning process. At the beginning of the experiment, the stimuli have no meaning or associated relevance, and such effects must be observable as differences in the slope of the amplitude change over time (trials) between relevance conditions.

However, after preregistering the study, we became confident that a more reliable and methodologically correct approach was necessary to test this hypothesis, compared to the one used in the preliminary analysis of the first sample.

For the preliminary analysis, trial-by-trial ERP amplitudes were averaged for the different relevance conditions using a moving window of 10 trials. The time period in which the relevance condition differed was determined based on non-overlapping 95% confidence intervals.

We acknowledged that more reliable results could be obtained by testing the interaction effect of relevance condition and trial on ERP amplitudes using linear

mixed models, similar to the ones used to test the other preregistered hypotheses. To obtain a sufficient reduction in the trial-by-trial variability due to noise in the data, trial-by-trial ERP amplitudes were still averaged along a moving window of consecutive trials, but the window size was increased to 20 trials. Furthermore, we realized that trial numbers were incorrectly coded during the preliminary analysis. Specifically, trials were numbered sequentially after the trial rejection step of data preprocessing. For example, if trial 3 was removed during preprocessing from the original sequence of trials 1, 2, 3, and 4, the resulting sequence of trials 1, 2, 4 was recoded as 1, 2, 3, respectively. This may have led to artificially higher or lower slopes of amplitude changes across trials. The manuscript describes an analysis that addresses an issue by correctly maintaining the original trial number before trial rejection.

Although we were confident in maintaining the preregistered hypothesis 7 based on the conceptual reasons described above, we excluded any evidence obtained from the preliminary analysis of the first sample of 24 participants. This decision was made because the new analysis approach would provide more reliable results. Given these changes, dividing the dataset into two separate samples would have been arbitrary and would have reduced the statistical power of the analysis.

Finally, we deviated from the preregistered analysis plan to the extent that we did not use dependent sample t-tests to analyze differences in ERP amplitudes between individual levels of relevance and font congruence in the test phase. As the linear mixed models to test the effects of relevance, font congruence, and their interaction were fitted in R (see Methods section in the main text), a reliable test of the significance of each level of the two factors and their interaction could be obtained

using the Satterthwaite approximation (Luke, 2017), via the function lmer from the lmerTest package (version 3.1-3; Kuznetsova et al., 2017).

S3. Analysis of the EPN component in the 200 – 300 ms time-window

In addition to the original time-window of 250 – 300 ms, EPN component was further analyzed between 200 and 300 ms over the same occipito-parietal cluster (O1, O2, P9, P10, PO7 and PO8).

Effects of monetary outcome on mean ERP amplitudes during the learning phase, interaction effects of monetary outcome and trial on ERP amplitudes during the learning phase, and interaction effects of monetary outcome and font congruence during the test phase were analyzed using the same procedure as for the original time-window, as described in the main text.

S3.1 Effects of monetary outcome on mean ERP amplitudes

Loss-associated stimuli also resulted in more negative **EPN** amplitudes compared to both zero-outcome and gain-associated stimuli (β = -0.41, *SE* = 0.15, *p* = .006, and, β = -0.31, *SE* = 0.14, *p* = .026, respectively). Gain associations did not result in significant EPN modulations compared to zero-outcome stimuli (**Table S1**).

S3.2 Temporal development of relevance effects across learning

No significant interaction effect of associated monetary outcome and trial number was observed on the EPN component (p > .05).

S3.3 Effects of associated monetary outcome and font congruence on mean ERP amplitudes

Likelihood ratio tests revealed significance for the full-null model ($\chi 2$ = 17.85, *df* = 5, *p* = .003). However, the EPN component amplitudes were not significantly modulated by the interaction effect of outcome category and font congruence (all *p*s > .05).

The reduced model without the interaction term showed significantly more negative amplitudes for gain- and loss-associated pseudowords compared to zero-outcome stimuli (β = -0.24, *SE* = 0.12, *p* = .038, and, β = -0.40, *SE* = 0.12, *p* = .002,

respectively). In addition, EPN amplitudes were significantly more negative for pseudowords presented in the incongruent than in the congruent font (β = -0.24, *SE* = 0.09, *p* = .013; **Table S2**).

Table S1. *Summary of the LMM Regarding the Effect of Outcome Association on the EPN Amplitudes in the 200 – 300 ms Time-Window during the Learning Phase*

Predictors	R2	Estimate	std. Error	95% CI	t value	p
**EPN (200 – 300 ms)**						
	.001					
Intercept		-2.53	0.33	-2.83 1.50		(1)
outcome (gain) (2)		-0.09	0.14	-3.24 2.47	-0.66	0.516
outcome (loss) (2)		-0.41	0.15	-2.95 2.84	-2.83	0.006

(1) not shown because of being of very limited interpretability

(2) comparison with the reference level (zero outcome)

Table S2. *Summary of the LMM Regarding the Effect of Outcome Association and Font Congruence on the EPN Amplitudes in the 200 – 300 ms Time-Window during the Test Phase*

Predictors	R2	Estimate	std. Error	95% CI	t	p
**EPN (200 – 300 ms)**						
	.008					
Intercept		-2.55	0.35	-3.25 -1.89		(1)
outcome (gain) (2)		-0.38	0.17	-0.70 -0.07	-2.32	0.023
outcome (loss) (2)		-0.49	0.16	-0.81 -0.19	-3.03	0.003
congruence (incongruence)		-0.39	0.16	-0.69 -0.09	-2.54	0.013
outcome (gain) (2) * congruence (incongr.)		0.29	0.22	-0.11 0.69	1.34	0.188
outcome (loss) (2) * congruence (incongr.)		0.18	0.22	-0.23 0.57	0.83	0.415

(1) not shown because of being of very limited interpretability

(2) comparison with the reference level (zero outcome)
